# Pulmonary Arterial Hypertension (PAH) as the Initial Manifestation of Systematic Lupus Erythematosus (SLE): A Rare Presentation

**DOI:** 10.7759/cureus.39359

**Published:** 2023-05-22

**Authors:** Sangita D Kamath, Tauheed Ahmed, Ajatshatru Upadhyay, Vijay Agarwal

**Affiliations:** 1 General Medicine, Tata Main Hospital, Jamshedpur, IND

**Keywords:** thrombosis, anti-phospholipids, lupus, hypertension, pulmonary

## Abstract

Pulmonary arterial hypertension (PAH) is an uncommon manifestation of systemic lupus erythematosus (SLE), affecting about 0.5% to 23.3% of the population worldwide. The causes of PAH associated with SLE are multifactorial. While it is generally associated with a full-blown picture of SLE, it may rarely be the presenting manifestation of the disease. We describe the case of a middle-aged woman who presented with features of severe PAH due to SLE. She was treated with vasodilators and immunosuppression (steroids and mycophenolate mofetil), with a partial response to treatment at six months follow-up.

## Introduction

Pulmonary manifestations of systemic lupus erythematosus (SLE) include pleuritis, pleural effusion, capillaritis with diffuse alveolar hemorrhage (DAH), shrinking lung syndrome, interstitial lung disease (ILD), pulmonary thromboembolism, and pulmonary hypertension (PH) [1]. Pulmonary arterial hypertension (PAH) is defined as a mean pulmonary arterial pressure of 20 mm Hg or higher by right heart catheterization at rest [2,3]. PH caused by SLE-related histopathological changes in the pulmonary vasculature is described as pre-capillary pulmonary arterial hypertension (PAH) and is classified as group I PH as per the World Health Organization (WHO), 4th World Symposium Clinical Classification of Pulmonary Hypertension, Dana Point, 2008 [1].

PAH associated with SLE (SLE-PAH) is a rare condition, and the diagnosis of this life-threatening complication is often delayed due to its rarity [1]. About 40% of SLE patients with early PAH remain asymptomatic [4], while others have varied and non-specific symptoms like dysp­nea, fatigue, chest pain, nonproductive cough, etc. These may mimic other, more frequent types of SLE-related pulmonary involvement, like pleural or pericardial effusion or ILD [3].

Here, we describe a rare case of a middle-aged female who presented with symptoms of severe PAH as the presenting manifestation of SLE with secondary antiphospholipid antibody syndrome (APS).

## Case presentation

A lady in her forties was admitted to our hospital with a history of graying of vision, followed by loss of consciousness and involuntary passage of urine in clothes. She regained consciousness within three minutes, and she appeared normal after the episode. She had two such episodes on the day of admission and five to six such episodes in the past three months. These episodes were especially related to exertion. Her symptoms initially began with exertional breathlessness, which started three years ago, and had gradually progressed from NYHA (New York Heart Association) class I to class III. There was a rapid progression of dyspnea from class III to class IV in the last month, and presently, she is dyspneic while doing activities of daily living like bathing, going to the washroom, and even at rest. There was a history of photosensitivity of the skin over the face and intermittent arthralgias of the small joints of the fingers of the hands and wrists over the past six months. She also noticed increased hair loss and a rash over the forehead and the upper back that was itchy and associated with a burning sensation during the same period. Over the last year, she has also complained of discoloration of her fingers and the tip of her nose when exposed to cold weather. However, before her current admission, she had not sought medical help for her symptoms. She was married and had a daughter. There was a history of two first-trimester abortions-the first in the eighth week and the second in the twelfth week of pregnancy. The third pregnancy was full-term with normal vaginal delivery. There was no history of other significant medical ailments or the use of illicit drugs.

**Figure 1:**
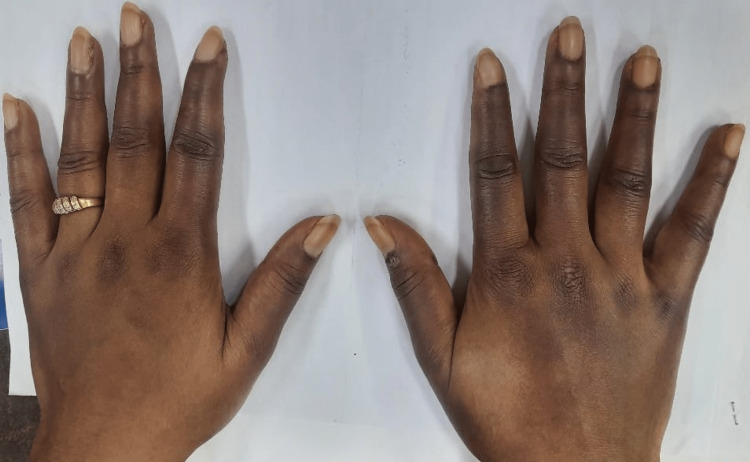
Sausage-shaped swelling of the fingers of both hands and knuckle hyperpigmentation

On general examination, she was coherent and had peripheral cyanosis (of fingertips and lips) and sausage-shaped swelling of the fingers (Figure 1).

There was no icterus, clubbing, lymphadenopathy, edema, or large joint swellings. There was an itchy, macular rash over the forehead and over the upper back. She was afebrile, her pulse rate was 98/minute, regular, low volume, all peripheral pulses felt equally on both sides, her blood pressure was 104/86 mm Hg in both arms, and her respiratory rate was 22/minute, thoracoabdominal, and regular. Examination of the cardiovascular system revealed a grade three parasternal heave and a loud pulmonary component of the second heart sound. The rest of the examination was normal. Examination of other systems was within normal limits. Other than mild thrombocytopenia (platelet count: 98,000/cu mm) and dyslipidemia, the rest of the basic blood investigations were normal. The urine examination was normal.

**Figure 2:**
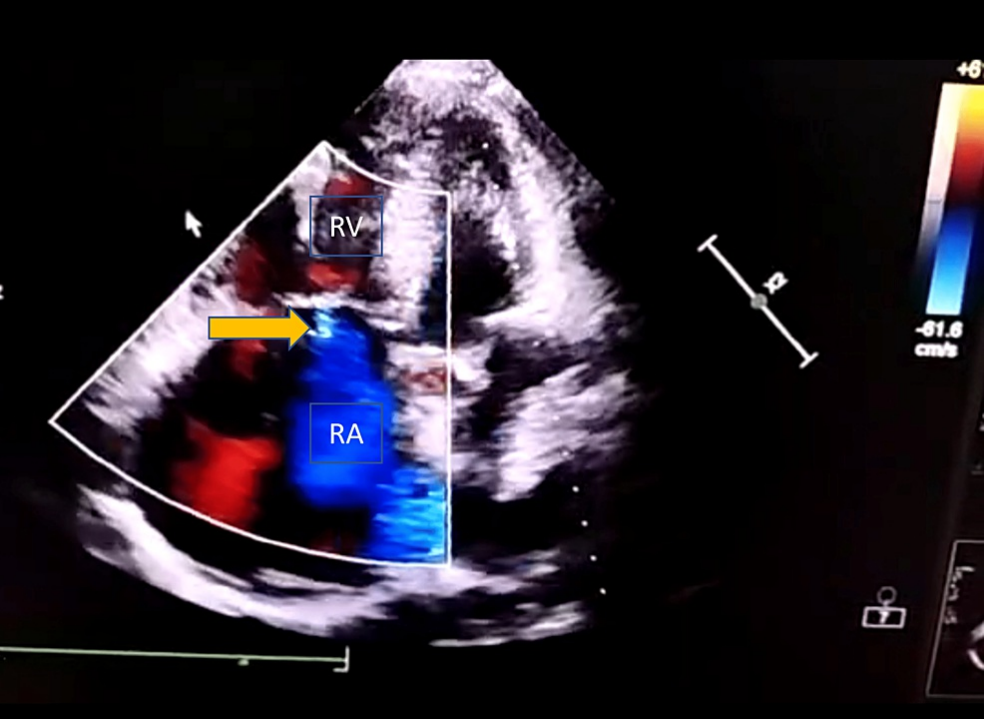
Echocardiography, four chamber view showing dilated right atrium (RA), right ventricle (RV), and tricuspid regurgitation (TR) jet (yellow arrow)

**Figure 3:**
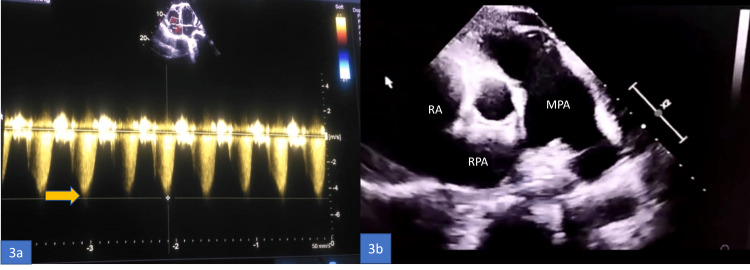
3a: Color doppler echocardiography showing severe tricuspid regurgitation (TR), tricuspid velocity jet of 4.3m/s (yellow arrow), secondary to severe pulmonary arterial hypertension. 3b: Echocardiography parasternal short-axis view–pulmonary artery bifurcation view (PSAX - PABV) showing dilated main pulmonary artery and its branches

Her chest radiograph revealed mild cardiomegaly. The electrocardiogram (ECG) showed sinus tachycardia, a right-ward axis, poor R wave progression from V1 to V4, and an S1Q3T3 pattern. Her trans-thoracic echocardiogram (TTE) showed an enlarged right atrium (RA) and right ventricle (RV), severe pulmonary arterial hypertension (PAH), an estimated pulmonary artery systolic pressure (PASP) of 95 mm Hg, moderate tricuspid regurgitation, and a left ventricular ejection fraction (LVEF) of 65% (Figures 2, 3a, 3b).

Contrast-enhanced computerized tomography (CECT) of the thorax showed normal lung parenchyma. Spiral computerized tomography (CT) angiography of the pulmonary artery, done to rule out chronic pulmonary thromboembolism (CPTE) as a cause of PAH, was normal except for dilated main pulmonary artery and right ventricle. Right heart catheterization was not done as the patient refused the same. Her six-minute walk test (6MWT) was limited to less than 50 meters due to dyspnea and oxygen saturation (SaO2) falling to less than 90%. Her hormonal and immunological assays were as in Table 1.

**Table 1:** Lab investigations (hormonal, vitamin, and antibody assays) ANCA: anti-cytoplasmic antibody, TSH: thyroid stimulating hormone, AMA: anti-mitochondrial antibody, HCV: hepatitis C virus, HBsAg: hepatitis B surface antigen, HIV: human immunodeficiency virus

Investigations	Values	Normal range
Antibodies to antinuclear antigen (ANA) - Hep-2	Positive	-
Antibodies to double stranded DNA (dsDNA) - ELISA	Negative	-
Serum C_3_ complement (mg/dl)	119.80	82—160
Serum C_4_ complement (mg/dl)	31.90	10—40
Anti Smith antibody IgG (EIA) (U/ml)	7.37	< 20
P- ANCA (anti-myeloperoxidase) (U/ml)	2.23	< 9
C- ANCA (serine proteinase - PR_3_) (U/ml)	2.5	< 3.5
Lupus anticoagulant (LA)	Detected (>50 GPL)	
Beta_ 2_ glycoprotein 1 – IgM	3.97 SMU	< 20 SMU
Anti-cardiolipin antibody – IgM (MPL)	24.4	< 14
Anti-cardiolipin antibody – IgG (MPL)	3.5	
Rheumatoid factor (RA)	Not detected	-
Anti-citrullinated antibodies (anti CCP)	Not detected	-
Anti-thyroid peroxidase (TPO) antibodies (IU/L)	0.50	< 9
Serum TSH (µIU/L)	7.72	0.3-6.5
Anti-AMA M_2_ (ng/mL)	42.8	Nonpregnant females: 2 to 29
Hbsag	Not detected	-
Anti HCV antibodies	Not detected	-
Antibodies to HIV 1 and 11	Non-reactive	-
Serum Vitamin B_12 _(pg/ml)	60	180 – 914
Serum 25-hydroxy D_3 _(ng/ml)	6.64	10 – 50

**Figure 4:**
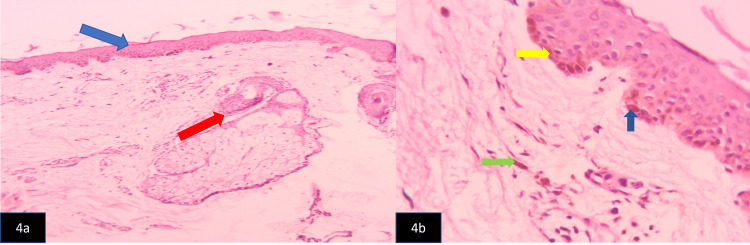
4a: Hematoxylin (H) and eosin (E) stained section x 100, photomicrograph of punch biopsy of forehead skin lesion showing perivascular and periadnexal chronic inflammatory cells (red arrow), thinned out squamous epithelium with keratin (blue arrow). 4b: H and E-stained section x 400, showing basal layer melanin pigmentation (yellow arrow), elongated and broadened Rete ridges (dark blue arrow), and melanin incontinence (green arrow).

Ultrasonography of the abdomen was normal. A punch skin biopsy from the forehead revealed normal squamous epithelium with mild hyperkeratosis, mildly elongated and broadened rete pegs, and basal melanocytes. There was mild perivascular lymphocytic infiltration and a periadnexal lymphoid infiltrate in the subepithelial dermis with focal melanin incontinence. There was no evidence of granulomatous inflammation or malignancy. These findings were suggestive of cutaneous manifestations of connective tissue disorder (CTD) (Figures 4a, 4b). 

**Figure 5:**
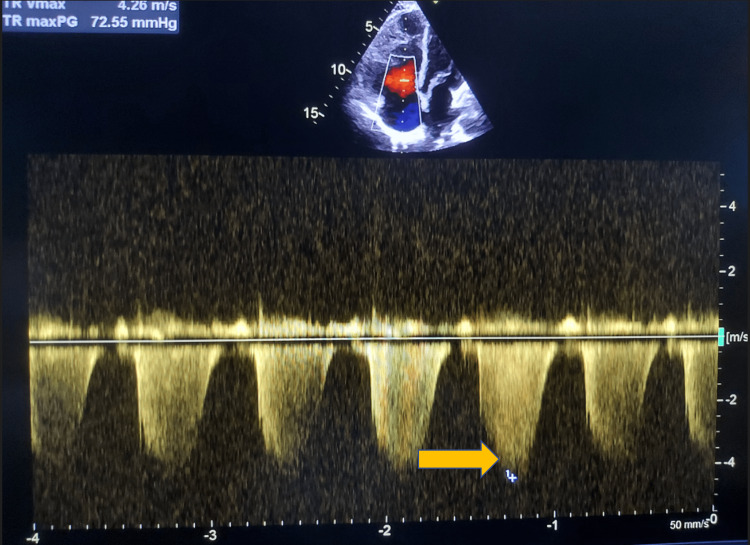
Color doppler echocardiography shows a decrease in TR velocity jet (yellow arrow) after six months of treatment TR: tricuspid regurgitation

Our patient was ANA-positive but negative for dsDNA and anti-Smith antibodies. She had arthralgias, non-scarring alopecia, intermittent oral ulcerations, skin lesions of lupus, thrombocytopenia, and Raynaud’s phenomenon. As she had more than four clinical criteria and one immunological criterion positive for lupus by Systemic Lupus International Collaborating Clinics (SLICC), she was diagnosed with SLE. In view of her history of recurrent first-trimester abortions and positive anti-phospholipid antibodies (IgM) on two occasions, 12 weeks apart, and evidence of SLE, she was diagnosed to have secondary anti-phospholipid antibody syndrome (APS) with severe primary pulmonary hypertension with functional New York Heart Association (NYHA) class lV and severe B12 deficiency. She was treated with oral aspirin 75 mg once daily, oral tadalafil 20 mg once daily for one week, later increased to 40 mg once daily, oral bosentan 62.5 mg twice daily, oral diltiazem 60 mg thrice daily, mycophenolate mofetil 500mg twice daily, hydroxychloroquine 400 mg once daily, rosuvastatin 10 mg with ezetimibe 10 mg for dyslipidemia, oral prednisolone 60 mg daily, gradually tapered by 5 mg per week after the initial four weeks, to presently 10 mg daily, oral B12 after the initial intramuscular B12 injections for seven days, and vitamin D supplements. At six months follow-up, she is symptomatically better. Her NYHA class of dyspnea improved from IV to II. Her repeat echocardiography showed a decrease in pulmonary arterial (PA) pressure to 72 mm Hg by TTE (Figure 5). 

## Discussion

Our patient presented with recurrent syncopal episodes, the cause of which, from the clinical history and examination, appeared to be cardiac. The differential diagnoses then considered were primary pulmonary hypertension (PPH), silent mitral stenosis (MS), and atrial septal defect (ASD) with an intermittent right-to-left shunt. Echocardiography findings were suggestive of severe PAH without a shunt and left heart disease. Hence, in the background of SLE with secondary APS and normal CT angiography of PA, a final diagnosis of SLE-associated severe PAH was made.

PAH is a well-recognized complication of SLE and is an important cause of morbidity and mortality after infection and organ failure [5]. According to the WHO classification, SLE-PAH is included in group I PH, namely connective tissue disease (CTD)-associated PAH (CTD-PAH) [2]. Earlier retrospective studies had reported the prevalence of PAH in SLE to be 0.5% to 6% [3], while in a systematic review by Xia YK et al., in the Chinese population, it was 2.8% to 23.3% [6]. The prevalence is higher in Asia than in Western countries [3]. This variation in the reported prevalence may be related to the different methods used to diagnose PAH.

A review of the published literature reveals that PAH sets in after a mean of 3.2 years after the diagnosis of SLE [7]. Once established, it progresses rapidly and usually carries a poor prognosis, with a 2-to 5-year survival rate after the initial diagnosis [6]. Our case was unique in the sense that the diagnosis of SLE was not established at the time of presentation. PH was the initial presenting feature that brought the patient to the clinician. A case akin to ours was published by Prete M et al. [3]. In their case, a 32-year-old patient presented with severe PAH (72 mm Hg) as the initial manifestation of SLE. She also had pericardial effusion and interstitial lung infiltrates and was successfully treated with mycophenolate mofetil and cyclo­sporine as first-line therapy, with reversal of PAH to 45 mm Hg at one year of diagnosis. She, however, did not have secondary APS.

Yet another case reported by Kawamura N et al. also had severe PAH associated with minimal lupus activity without arthritis, renal, neurological, or pulmonary involvement [8]. This patient had progressive PAH with no response to chronic epoprostenol therapy. A lung biopsy showed only intimal thickening of pulmonary arterioles without lung parenchymal changes.

The pathogenesis of PAH in SLE has not been well established. Autoimmune vasculitides with endothelial dysfunction (mediated by anti-endothelial cell antibodies [AECA]), abnormal vasospasm, high circulating levels of vasoconstrictors, in-situ thrombosis, and thromboembolism related to antiphospholipid syndrome (APS) have all been implicated [3,6].

SLE may be associated with secondary APS syndrome, as in our case. The presence of aPL antibodies has been linked to the development of PAH. 51.3% (46.2 to 56.8%) of lupus patients with PAH had anticardiolipin antibodies versus 23.8% (16 to 28.9%) of SLE patients without PAH in the Chinese population [6]. In the same study, the incidence of PAH was found to be higher in those SLE patients who had anti-beta2 GPI antibodies as compared to those who did not (25% versus 12%) [6]. A meta-analysis of 31 studies involving 4,480 patients with SLE by Zuily S et al. showed that lupus patients with positive aPL antibodies had a higher prevalence of PAH (12.3%) as compared with those without antibodies (7.3%) (OR: 2.28, 95% CI, 1.65 to 3.15; p < 0.0001) [9].

The role of aPL in the pathogenesis of PAH is unclear, but there is evidence that aPL may directly trigger inflammation and cause both platelet and endothelial activation, thus triggering thrombosis and chronic thromboembolic pulmonary hypertension (CTEPH) - WHO group 4 [2]. Increased levels of circulating endothelin-1 (ET-1) are observed in patients with aPL antibodies [10]. Further, aPLs have been reported to directly induce the proliferation of vascular cells in the intima and media, leading to non-thrombotic vasculopathy [7].

Left heart valvulopathy involving mitral and aortic valves (Libman - Sack’s endocarditis, resulting in thickening, vegetation, and valvular dysfunction) has been described in patients with SLE [11]. Valvular damage then causes regurgitation and transmission of back pressure, resulting in pulmonary venous hypertension (PVH - classified as WHO Group 2 PH [2]. SLE may also be associated with interstitial lung disease (ILD), which can result in PH (WHO Group 3 PH) [1,2]. Our patient had a normal high-resolution CT (HRCT) thorax and normal aortic and mitral valves, thus ruling out these causes of PAH.

As our patient was at an increased risk of thrombotic arteriopathy, we did a CT angiography of the pulmonary artery, which was normal, thus excluding pulmonary embolism. Thus, there was no reason to explain her PAH other than SLE-related inflammatory vasculitis. Some of the factors predicting the risk of developing PAH in SLE include high levels of endothelin 1, selectin, anti-cardiolipin, and anti-U1-RNP antibodies, as well as Raynaud’s phenomenon [3,6]. 41.4% of the patients with SLE and PAH had Raynaud’s phenomenon in the review by Xia YK et al., suggesting the role of vasospasm in PAH [6]. This was also seen in our case and could reflect underlying generalized vasoconstriction.

Her serum anti-AMA M2 was strongly positive at high titers. Anti-AMA M2 is specific for primary biliary cholangitis (PBC). However, till the present admission, the patient did not have clinical features of PCB and needed follow-up. Her liver enzymes were normal. Rarely there can be false-positive elevations of AMA M2 in patients with rheumatoid arthritis, syphilis, sarcoid, and acute hepatitis A [12].

The treatment of PAH associated with SLE includes targeted therapy with vasodilators (endothelin receptor antagonists, phosphodiesterase type-5 inhibitors, prostacyclin analogs, soluble guanylate cyclase inhibitors, and calcium channel blockers), steroids, immunosuppression, anticoagulants in cases of chronic thromboembolism [2]. No single group of therapeutic agents is fully effective [13]. We did not start anticoagulation in our patient as there was no evidence of CTEPH, and anti-cardiolipin IgM antibodies were present in low titers. So, our patient was put on vasodilators and immunosuppression (steroid and mycophenolate), with partial resolution of PAH by six months. Earlier literature has reported exacerbations of PAH during episodes of SLE flares and, hence, responsiveness to intensive immunosuppression with steroids and cyclophosphamide in addition to vasodilators [14]. However, more randomized studies are needed to confirm this.

Though our patient had suffered from arthritis and a skin rash for six months and Raynaud’s phenomenon for one year, she had taken only symptomatic treatment and had not visited a clinician. Through this case, we wish to highlight this rare presentation of SLE, as early diagnosis may halt the progress of this otherwise relentless complication. Echocardiography provides a simple, bedside, non-invasive screening method.

## Conclusions

PAH is a well-recognized complication of SLE and carries high morbidity. The cause of PAH in SLE is multifactorial and needs a thorough evaluation. Patients with APL antibodies are at high risk of developing PAH. Though the development of PAH correlates with disease severity, rarely, PAH may be the initial manifestation of SLE, as in our case. Severe PAH must be considered a cause of progressive dyspnea, and clinicians must have a high index of suspicion for screening these patients for PAH, as early treatment may reduce the morbidity.
